# Reports of Batch-Dependent Suspected Adverse Events of the BNT162b2 mRNA COVID-19 Vaccine: Comparison of Results from Denmark and Sweden

**DOI:** 10.3390/medicina60081343

**Published:** 2024-08-19

**Authors:** Vibeke Manniche, Max Schmeling, Jonathan D. Gilthorpe, Peter Riis Hansen

**Affiliations:** 1 LIVA, Rosenvængets Allé 6A, DK-2100 Copenhagen, Denmark; vibeke@vibekemanniche.dk; 2 Innometric, Bavnehøjvej 5, Hellum, DK-9520 Skørping, Denmark; max@schmeling.dk; 3 Department of Medical and Translational Biology, Umeå University, 901 87 Umeå, Sweden; jonathan.gilthorpe@umu.se; 4 Department of Cardiology, Copenhagen University Hospital—Herlev and Gentofte, Gentofte Hospitalsvej 1, DK-2900 Hellerup, Denmark; 5 Department of Clinical Medicine, Faculty of Health and Medical Sciences, University of Copenhagen, Blegdamsvej 3B, DK-2200 Copenhagen, Denmark

**Keywords:** COVID-19, BNT162b2, vaccine, safety, adverse events

## Abstract

*Background and Objective*: An unexpected batch-dependent safety signal for the BNT162b2 mRNA COVID-19 vaccine was recently identified in a nationwide study from Denmark, but the generalizability of this finding is unknown. Therefore, we compared batch-dependent rates of suspected adverse events (SAEs) reported to national authorities in Denmark and Sweden. *Materials and Methods*: SAE and vaccine batch data were received from national authorities in Denmark and Sweden, and analyses of heterogeneity in the relationship between numbers of vaccine doses and SAEs per batch were performed, along with comparison of SAE rates and severities for batches that were shared between the two countries. *Results*: Significant batch-dependent heterogeneity was found in the number of SAEs per 1000 doses for both countries, with batches associated with high SAE rates detected in the early phase of the vaccination campaign and positive correlations observed between the two countries for the severity of SAEs from vaccine batches that they shared. Mild SAEs predominated in the batches used in the early part of the vaccination roll-out, where markedly higher SAE rates per 1000 doses in Denmark for the batches that were shared between the two countries suggested that a large proportion of these SAEs were under-reported in Sweden. *Conclusions*: The batch-dependent safety signal observed in Denmark and now confirmed in Sweden suggests that early commercial batches of BNT162b2 may have differed from those used later on, and these preliminary and hypothesis-generating results warrant further study.

## 1. Introduction

The critical appraisal of data from the global emergency vaccination program against COVID-19 has identified concerns about risks associated with the predominant COVID-19 mRNA–lipid nanoparticle vaccine platform [1,2]. For example, secondary analyses of the pivotal placebo-controlled randomized trials indicated an excess of severe adverse events and irregularities in data integrity and regulatory oversight of the BNT162b2 vaccine (Comirnaty, Pfizer-BioNTech) trials [3,4]. Moreover, data from Pfizer showed that manufacturing changes were made during upscaling from clinical to commercial vaccine batches, and leaked documents from the European Medicines Agency (EMA) suggested that early commercial BNT162b2 vaccine batches contained unexpectedly low amounts of intact mRNA [1,2,5]. Along this line, the first Periodic Safety Update Report (PSUR) for the BNT162b2 vaccine submitted to EMA by the market authorization holder (BioNTech) on 19 August 2021 suggested considerable variation in the numbers of SAEs between different BNT162b2 batches administered during the early vaccination campaign [6].

We recently reported the results of a nationwide study from Denmark indicating a batch-dependent safety signal for the BNT162b2 vaccine, with unexpected heterogeneity in the relationship between the rates of reported suspected adverse events (SAEs) per 1000 doses and the number of doses in individual vaccine batches [7]. Three distinct clusters of BNT162b2 vaccine batches were identified, with highly variable SAE reporting rates. Also, a temporal reduction in batch-dependent SAE rates was found that paralleled the temporal sequence of vaccine batch roll-out, with batches administered at the beginning of the vaccination campaign displaying disproportionally high SAE rates [7,8]. Interestingly, the batches with the highest SAE rates were also among those reported to have high numbers of SAEs in the PSUR from the market authorization holder [6]. However, these hypothesis-generating results called for validation, preferably in a country with comparable demographics and where vaccine batches exhibited overlap with those distributed in Denmark. Sweden and Denmark are proximate Scandinavian countries that shared several BNT162b2 vaccine batches, albeit they otherwise displayed somewhat different responses to the COVID-19 pandemic, with extensive use of authoritative politics-led regulatory instruments in Denmark compared to Sweden, which exhibited expert-led management and abstained from imposing a national lockdown [9]. Therefore, we compared BNT162b2 batch-dependent SAE rates in Denmark and Sweden, including in these analyses also newly obtained data from Denmark that had not been processed by the Danish regulatory authorities at the time of our previous report [7].

## 2. Materials and Methods

### Data Sources

The Danish BNT162b2 data covering a period from the start of the vaccination campaign (27 December 2020) to 5 October 2023 were received on request from the Danish Medical Agency (DKMA) on 6 October 2023. Notably, the current data from the DKMA differed from our previously published Danish study by including a backlog of a total of 49,749 SAEs that had not been processed by the DKMA at the time of our earlier report [7]. The SAE reporting system run by the DKMA is a spontaneous passive surveillance system that receives SAE reports from any source, e.g., patients, healthcare workers, and other members of the public [10]. Summary demographics (sex, age, and status as a private citizen or healthcare worker) of the SAE registrants were obtained. The data were classified by the DKMA according to SAE severity (mild, severe [hospitalization, life-threatening illness, permanent disability or congenital malformation], or SAE-related death, respectively) and included 34,410 registered and processed reports, with a total of 93,245 SAEs or 2.71 SAEs per report. The vaccine batch label codes, which were usually two capital letters followed by four numbers, e.g., ‘FE2090’, and displayed a time-dependent alpha-numerical progression throughout vaccine roll-out, were incomplete or missing for 19.2% of reported SAEs, and these batches were not used in the analyses. The number of doses per vaccine batch shipped from the Danish State Serum Institute (SSI) until 28 March 2023 were obtained on request on this date, by which time 72 separate BNT162b2 batches had been distributed by the SSI. Since data on the number of doses administered per individual batch during the latter part of the examined period were not made available to us by the SSI despite repeated requests, we limited the current direct comparison with data from Sweden to the 52 batches (including batches that were shared between the two countries) used in the period from 27 December 2020 to 11 January 2022, for which we previously found a < 0.15% difference between numbers of shipped and administered doses in Denmark [7,8]. During this period, 30,646 reports were registered and processed in Denmark, with a total of 83,667 SAEs, or 2.73 SAEs per report. 

For the Swedish BNT162b2 data, reported SAEs and the number of administered doses per vaccine batch from the start of the vaccination campaign on 27 December 2020 to 19 January 2024 were received on request from the Swedish Medical Products Agency (SMPA; Läkemedelsverket). The number of administered doses per vaccine batch was obtained from the Public Health Agency of Sweden (Folkhälsmyndigheten) on 29 January 2024. The SMPA-managed SAE reporting and data capture system is a passive surveillance reporting system akin to the DKMA-managed system [11]. The Swedish SAE data included 56,784 registered and processed reports, with a total of 219,731 SAEs, or 3.87 SAEs per report. At the time of data retrieval, only 401 reports from the accessed period had not been processed and 4.4% of SAE registrations did not include details of the vaccine batch, and these incomplete records were not used in the cluster analyses. During the period, doses from 112 separate BNT162b2 vaccine batches were utilized, and out of a total of 22,333,887 administered doses, 10,780 (0.05%) doses did not have a valid batch label code and were not included in the analyses. The period from 27 December 2020 to 11 January 2022 included 48,172 registered and processed reports, with a total of 186,672 SAEs, or 3.88 SAEs per report.

## 3. Statistical Analysis

All data were received in tabular format and registered on an SAE level. Each SAE was registered separately, and in case several SAEs were reported by an individual, these were registered on separate SAE lines. The batch label code of a vaccine dose with the reported SAEs was recorded on respective SAE lines, so that data were organized to allow for the determination of SAEs on a batch level. The rate of SAEs per 1000 doses for each individual batch was calculated by dividing the total number of SAEs registered by the number of administered doses from the respective batches. Analysis of heterogeneity in the relationship between the number of doses and number of SAEs per batch was performed by normalizing SAE rates per batch by logarithmic transformation, followed first by hierarchical cluster analysis to determine the appropriate number of clusters and second by nonhierarchical cluster analysis, after which the resulting batch clusters were tested for significant differences using ANOVA (the general linear model [GLM]), as undertaken previously [7,8]. This approach was selected since all modeled relationships (trendlines) between SAEs and dose numbers were assumed to be linear and passed through the graphical origin point (0,0), as there was invariably 0 SAEs with 0 vaccine doses. Heterogeneity was hereby reduced to one dimension only (the SAE rate), which was segmented using nonhierarchical cluster analysis. For the cluster analyses, data from 27 December 2020 to 11 January 2022 and from 27 December to 19 January 2024 were used for Denmark and Sweden, respectively. For comparison of SAEs for batches used in the period from 27 December 2020 to 11 January 2022 in the two countries, batches were organized according to the alpha-numerical progression of batch label codes, apart from the few batches that did not comply with the alpha-numerical code label and which were, therefore, placed according to the month of the peak number of administered doses from the respective batches. Thereafter, correlation analyses were performed between the rates of mild SAEs, severe SAEs, and SAE-related deaths, respectively, in the two countries for the 12 batches that were used in both Denmark and Sweden.

This study relied exclusively on publicly available anonymized data and was, therefore, not subject to research ethics board review. 

## 4. Results

Most reported SAEs were for women, who comprised approximately 70% and 75% of all SAE reports in Denmark and Sweden, respectively, during the study period. In both countries, women represented 85–90% of SAE reports in the early phase of BNT162b2 vaccine roll-out, where high SAE rate batches were apparent (see below). In Denmark, approximately 40% of all SAE reports were from healthcare workers, and this proportion was lower in Sweden, where healthcare workers submitted approximately 15% of reports. However, in both Denmark and Sweden, the percentages of SAE reports from healthcare workers were higher (approximately 50% and 30%, respectively) for the early administered vaccine batches compared with those administered later on. In both countries, a majority of persons (up to 90%) reporting SAEs with the early high SAE rate vaccine batches were <70 years of age, while the proportion of elderly people with reported SAEs rose sharply thereafter. 

### 4.1. Cluster Analyses of Heterogeneity in SAE Rates between BNT162b2 Batches

**Figure 1:**
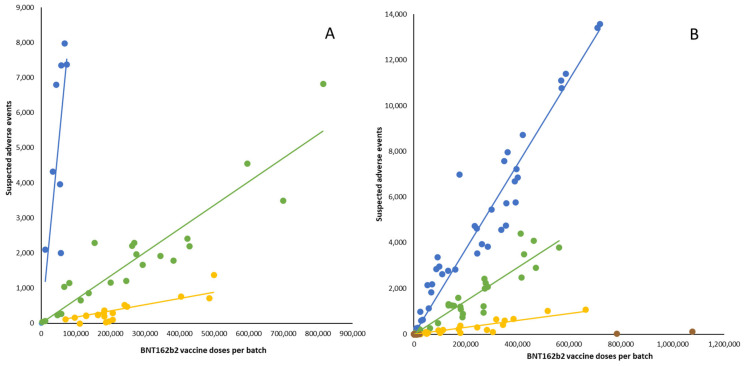
Number of suspected adverse events after BNT162b2 mRNA vaccination in Denmark in the period from 27 December 2020 to 11 January 2022 (**A**) and in Sweden in the period from 27 December 2020 to 19 January 2024 (**B**) according to the number of doses per batch. Each dot represents a single vaccine batch. Trendlines are linear regression lines from cluster analyses. (**A**): blue: R^2^ = 0.90, β = 0.1021 (confidence interval [CI] 0.0710–0.1332); green: R^2^ = 0.94, β = 0.0066 (CI 0.0058–0.0074); yellow: R^2^ = 0.86, β = 0.0018 (CI 0.0014–0.0021). (**B**): blue: R^2^ = 0.97, β = 0.0186 (CI 0.0175–0.0196); green: R^2^ = 0.94, β = 0.0073 (CI 0.0067–0.0079); yellow: R^2^ = 0.90, β = 0.0015 (CI 0.0013–0.0017).

The SAE data from Denmark during the period from 25 December 2020 to 11 January 2022 with the inclusion of a backlog of SAEs that had not been processed at the time of our previous report [7] demonstrated three distinct batch clusters with highly significant differences in SAE rate trendlines delineating high, moderate, and low SAE rate batches, respectively (Figure 1A). The temporal sequence of these batch clusters was in overall accord with the course of vaccine roll-out determined by the alpha-numerical progression of batch label codes. The initial hierarchical cluster analysis of Swedish data from the full data retrieval period was also segregated into three predominant batch clusters comprising 40, 33, and 18 batches, respectively. However, owing to the non-negligible statistical influence of two additional smaller clusters, a model with four clusters was deemed most appropriate and showed highly significant differences between batch cluster trendlines (Figure 1B). Accordingly, the final four clusters comprised 40, 33, 23, and 16 vaccine batches, with the fourth cluster consisting of 16 batches mainly comprising batches with few doses administered during the late part of the study period. In addition to the increased inter-cluster heterogeneity observed for the Swedish data, the temporal evolution of the batch clusters was not entirely uniform in the two countries. Indeed, in Denmark, shifts from the high SAE rate (Figure 1, blue trendline) to the moderate SAE rate (Figure 1, green trendline) batches and from the moderate SAE rate to low SAE rate (Figure 1, yellow trendline) batches, respectively, occurred with the batch label codes ‘EM’-‘EP’ and ’FE’-‘FG’, respectively, while in Sweden, these transitions were observed later on, i.e., around batch label codes ‘FG’-‘FH’, ‘FN-FP’, and (for transition from yellow to brown trendline batches) ‘GJ-GL’, respectively.

### 4.2. Batch-Dependent SAE Rates for Vaccine Batches Shared between Denmark and Sweden

**Figure 2:**
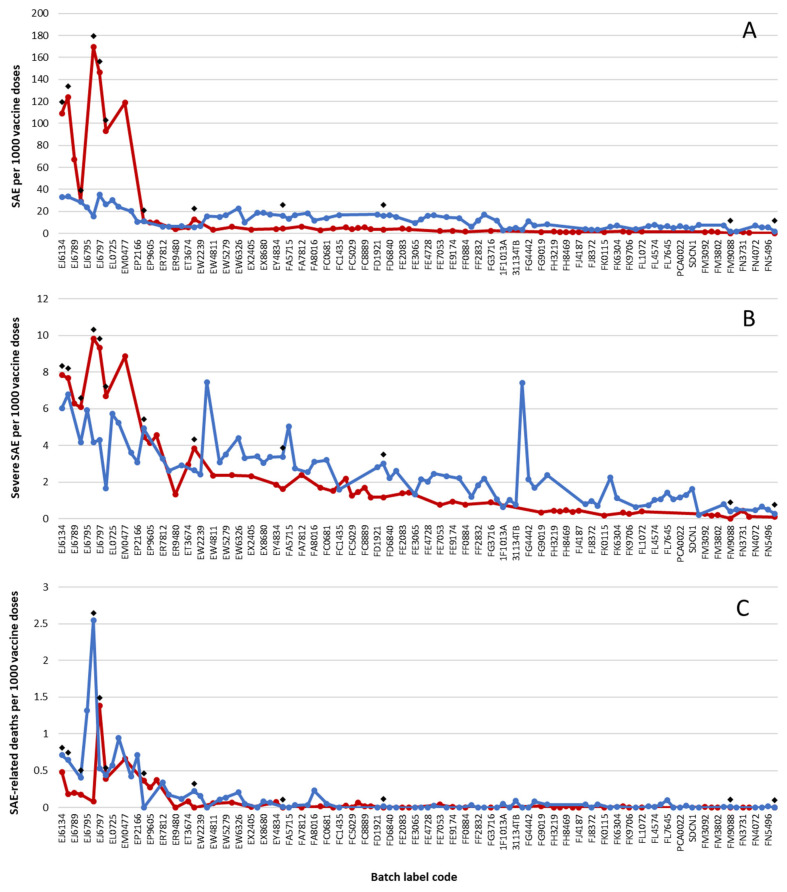
Suspected adverse reactions (SAEs) per 1000 doses from individual BNT162b2 batches administered in Denmark (red) and Sweden (blue) according to SAE severities reported for consecutive vaccine batches during vaccine roll-out from 27 December 2020 to 11 January 2022. Note a ten-fold increase in *y*-axis scales in (**A**–**C**). Batches were arranged consecutively according to the alpha-numerical progression of batch label codes and/or (for batches that did not conform with the standard two-letter-and-four-digit labeling scheme, e.g., ‘1F1013A’) the month where the peak number of doses was administered from the respective batches. Due to space constraints, not all batch labels codes are identified on the abscissa. Black diamonds represent batches that were shared between the two countries.

The SAE rates for consecutive batches were compared by matching the order of batches (reflecting their temporal roll-out) between the two countries, e.g., Danish batches ‘FE2083’ and ‘FE2090’ were placed in between Swedish batches ‘FD9309’ and ‘FE3065’ (Figure 2A–C). This organization of the data disclosed that batch-dependent rates of mild SAEs were markedly higher in Denmark for vaccine batches used early in the study period. This pattern was less pronounced for severe SAEs, and for SAE-related deaths, batch-dependent data were comparable between the two countries.

Twelve vaccine batches were administered in both countries, and for Denmark vs. Sweden, these shared batches represented 6 vs. 9 high-SAE-rate batches, 4 vs. 1 moderate-SAE-rate batches, and 2 vs. 2 low-SAE-rate batches, respectively. The correlation coefficients between the shared batches from the two countries were 0.682 for mild SAEs, 0.685 for severe SAEs, and 0.078 for SAE-related deaths, indicating a moderate-to-strong association for mild and severe SAEs, but a very low correlation for SAE-related deaths. The large (0–2.55) variation in reported SAE-related deaths per 1000 doses for the shared batches likely contributed to the observed very low correlation of these SAEs between the two countries, while the large decline in SAE rates per batch over time for subsequent batches probably added to the considerably stronger correlation between the batches for mild and severe SAEs, respectively.

## 5. Discussion

The present study extends our previous report on significant heterogeneity in BNT162b2 batch-dependent SAE rates for the BNT162b2 vaccine in Denmark by demonstrating a similar batch-dependent safety signal in nationwide data from Sweden. Both countries had high-SAE-rate batches in the early phase of vaccine roll-out, followed by declining rates of batch-dependent SAE rates over time, and summary demographic data did not provide any obvious explanation for these observations. Also, a positive correlation was observed between the two countries regarding the severities of SAEs from the batches that they shared. Notably, however, Danish batch-dependent SAE rates per 1000 doses exhibited somewhat more pronounced heterogeneity than the Swedish data, especially in the early phase of the vaccination campaign and with regard to mild SAEs, where markedly lower SAE rates were reported in Sweden for batches shared between the two countries. 

Retrospective and independent analyses have enabled a reappraisal of the risk/benefit ratio of COVID-19 mRNA vaccines, which has become subject to increasing debate. For example, excess risk of adverse events in BNT162b2 clinical trials has been suggested, and alterations in the vaccine product during upscaling of the manufacturing process may have occurred [1,2,3,4,5,6]. Numerous sources of bias influencing the estimated effectiveness of COVID-19 vaccines have also been identified that limit the interpretation of results of subsequent nonrandomized observational studies [12,13]. Along this line, the established system for the quality control of vaccines has been questioned, reinforcing calls for unconstrained, precise, and timely pharmacovigilance of vaccines from the point of production to the point of care, including both reactive and preventive measures with the use of SAE data on individual doses [14,15]. 

The current findings of a batch-dependent safety signal, in both Denmark and Sweden, are corroborated by the observed correlation between batch-dependent mild and severe SAEs for vaccine batches administered in both countries. The large majority of reported SAEs concerned women, and it is notable that in October 2022, the EMA requested that the product information of the BNT162b2 and mRNA-1273 (Spikevax, Moderna) mRNA vaccines was updated with information about heavy menstrual bleeding as a side effect, and an increased risk of unexpected vaginal bleeding in nonmenstruating women has been reported [16,17]. Although we did not have access to the clinical description of SAEs, it seems unlikely, however, that irregular vaginal bleeding alone would fully account for the higher percentage of reports concerning women, and more studies are required to understand why women may more frequently report and/or be affected by mRNA vaccine adverse effects.

A commercial vaccine product should be identical in all batches including those shared between countries, and it is surmised that vaccinated individuals in Denmark and Sweden objectively encountered the same rates of SAEs and had similar means and opportunity to report SAEs. Therefore, our data suggest that mild SAEs were markedly under-reported in Sweden during the early phase of vaccine roll-out. The factors that modulate the spontaneous reporting of SAEs on a population scale are multifactorial, but it is notable that the governmental response to the COVID-19 pandemic differed considerably in Denmark and Sweden, with a high hierarchical command and control governance in Denmark (including societal lockdown and selective engagement with healthcare expertise) and a network-based approach with extensive regional and local autonomy in Sweden [9]. For example, in June 2021, i.e., during the period of the administration of the ‘green’ moderate SAE rate batches, the DKMA issued a request for the public not to report simple and transient SAEs, whilst to our knowledge, similar regulatory pleas were not issued in Sweden. Altogether, the differing control governance arrangements in Denmark and Sweden might, therefore, have suggested that, contrary to our findings, the under-reporting of SAEs in the early phase of the vaccination campaign would be most likely in Denmark compared with Sweden. However, we also observed that during the study period, a considerably higher percentage of SAE reports (40 vs. 15%) were from healthcare workers rather than private individuals in Denmark compared to Sweden. Interestingly, periodic yearly reports from the SMPA summarizing SAE reports for all marketed pharmaceutical products in Sweden have shown a distinct shift in ratios of SAE reports from healthcare workers/the public in 2021, with ratios of 72/28% in 2020 and 53/47% in 2021, respectively, with this shift mainly determined by changes in reporting ratios for the COVID-19 vaccines [18,19]. Whether healthcare workers in Sweden were less inclined to report SAEs from the BNT162b2 vaccine in Sweden requires further study, including to determine the potential role of the perceived risk that such reports may be linked to repressive measures from governmental and regional health authorities [20]. Notably, the importance of political observance for the reporting of SAEs related to COVID-19 vaccines was recently suggested in a study from the US Vaccine Adverse Events Reporting System (VAERS), a passive surveillance system like the reporting systems managed by the DKMA and SMPA, which showed that the more likely it was that US states voted Republican, the more likely these states were to report vaccine-related SAEs [21]. In any case, the under-reporting of data that best represent objective rates of SAEs may have important public health consequences, e.g., by impeding long-term follow-up of subjects with SAEs and reducing the likelihood of backtracking later clinical events to SAEs. Also, it seems plausible that if short- and medium-term SAEs are under-reported, then long-term SAEs are even less likely to be reported.

The current validation by Swedish data of the batch-dependent safety signal reported from Denmark adds weight to the hypothesis that early commercial BNT162b2 vaccine batches may have differed from the latter batches and that batch-level product quality surveillance and pharmacovigilance may have been suboptimal during the BNT162b2 vaccine roll-out. However, we emphasize that our results are preliminary and hypothesis-generating, given the inherent limitations of spontaneous SAE reporting systems like the DKMA and SMPA. Notably, such passive reporting systems may capture < 15% of SAEs [22]. Also, the current data were incomplete and subject to variable quality information, and factors such as vaccine efficacy, pre-existing immunity and booster schedules, and clinical details and long-term effects of reported SAEs were not examined. 

## 6. Conclusions

The batch-dependent safety signal observed in Denmark and now confirmed in Sweden, may suggest that early commercial batches of BNT162b2 may have differed from those used later on, and these preliminary and hypothesis-generating results call for further studies of their causes and consequences.

## Data Availability

The raw data supporting the conclusions of this article will be made available by the authors on request.

## References

[1] Rhodes P., Parry P. (2024). Gene-based COVID-19 vaccines: Australian perspectives in a corporate and global context. Pathol. Res. Pract..

[2] Igyártó B.Z., Qin Z. (2024). The mRNA-LNP vaccines—The good, the bad and the ugly?. Front. Immunol..

[3] Fraiman J., Erviti J., Jones M., Greenland S., Whelan P., Kaplan R.M., Doshi P. (2022). Serious adverse events of special interest following mRNA COVID-19 vaccination in randomized trials in adults. Vaccine.

[4] Thacker P.D. (2021). COVID-19: Researcher blows the whistle on data integrity issues in Pfizer’s vaccine trial. BMJ.

[5] Tinari S. (2021). The EMA COVID-19 data leak, and what it tells us about mRNA instability. BMJ.

[6] Periodic Safety Update Report #1 for COVID-19 Mrna Vaccine (Nucleoside Modified) (BNT162b2). Submitted to the European Medicines Agency on 19 August 2021, Made Publicly Available in Response to A Freedom of Information Act (FOIA) Request from an Anonymous Reader and Published by the Austrian Science and Political Blog, TKP. https://tkp.at/2023/01/17/aerzte-tragen-die-verantwortung-bei-impf-schaeden-und-fuer-deren-meldung.

[7] Schmeling M., Manniche V., Hansen P.R. (2023). Batch-dependent safety of the BNT162b2 mRNA COVID-19 vaccine. Eur. J. Clin. Investig..

[8] Schmeling M., Manniche V., Hansen P.R. (2023). Batch-dependent safety of the BNT162b2 mRNA COVID-19 vaccine. Eur. J. Clin. Investig..

[9] Christensen T., Jensen M.D., Kluth M., Kristinsson G.H., Lynggaard K., Lægreid P., Niemikari R., Pierre J., Raunio T., Adolf Skúlason G. (2023). The Nordic governments’ responses to the COVID-19 pandemic: A comparative study of variation in governance arrangements and regulatory instruments. Regul. Gov..

[10] Aagaard L., Stenver D.I., Hansen E.H. (2008). Structures and processes in spontaneous ADR reporting systems: A comparative study of Australia and Denmark. Pharm. World Sci..

[11] Kälkner K.M., Sundström A., Nurminen M.L., Larsson M., Ljung R., Arthurson V. (2023). Optimizing Safety Surveillance for COVID-19 vaccines at the Swedish Medical Products Agency. Drug Saf..

[12] Ioannidis J.P.A. (2022). Factors influencing estimated effectiveness of COVID-19 vaccines in non-randomised studies. BMJ Evid. Based Med..

[13] Fung K., Jones M., Doshi P. (2024). Sources of bias in observational studies of COVID-19 vaccine effectiveness. J. Eval. Clin. Pract..

[14] Bruce Yu B., Taraban M.B., Briggs K.T. (2021). All vials are not the same: Potential role of vaccine quality in vaccine adverse reactions. Vaccine.

[15] Bruce Yu B., Briggs K.T., Taraban M.B. (2023). Preventive Pharmacovigilance: Timely and precise prevention of adverse events through person-level patient screening and dose-level product surveillance. Pharm. Res..

[16] Meeting Highlights from the Pharmacovigilance Risk Assessment Committee (PRAC) 24–27 October 2022. https://www.ema.europa.eu/en/news/meeting-highlights-pharmacovigilance-risk-assessment-committee-prac-24-27-october-2022.

[17] Blix K., Laake I., Juvet L., Robertson A.H., Caspersen I.H., Mjaaland S., Skodvin S.N., Magnus P., Feiring B., Trogstad L. (2023). Unexpected vaginal bleeding and COVID-19 vaccination in nonmenstruating women. Sci. Adv..

[18] Medical Products Agency Annual Report for Suspected Side Effects 2020 [Årsrapport för Misstänkta Biverkningar 2020—Enheten för Läkemedelssäkerhet]. https://www.lakemedelsverket.se/4992f0/globalassets/dokument/publikationer/biverkningsrapporter/biverkningsrapportering-2020.pdf.

[19] Medical Products Agency Annual Report for Suspected Side Effects 2021 [Årsrapport för Misstänkta Biverkningar 2021—Enheten för Läkemedelssäkerhet]. https://www.lakemedelsverket.se/49e130/globalassets/dokument/publikationer/biverkningsrapporter/arsrapport-for-biverkningar-2021.pdf.

[20] Shir-Raz Y., Elisha E., Martin B., Ronel N., Guetzkow J. (2023). Censorship and suppression of COVID-19 heterodoxy: Tactics and counter-Tactics. Minerva.

[21] Asch D.A., Luo C., Chen Y. (2024). Reports of COVID-19 Vaccine Adverse Events in Predominantly Republican vs Democratic States. JAMA Netw. Open.

[22] Hazell L., Shakir S.A. (2006). Under-reporting of adverse drug reactions: A systematic review. Drug Saf..

